# *Maackia amurensis* seed lectin structure and sequence comparison with other *M. amurensis* lectins

**DOI:** 10.1016/j.jbc.2025.108466

**Published:** 2025-03-28

**Authors:** Ashok R. Nayak, Cayla J. Holdcraft, Ariel C. Yin, Rachel E. Nicoletto, Caifeng Zhao, Haiyan Zheng, Dmitry Temiakov, Gary S. Goldberg

**Affiliations:** 1 Biochemistry & Molecular Biology Department, Thomas Jefferson University, Philadelphia, Pennsylvania, USA; 2 Molecular Biology Department, Rowan Virtua SOM, Rowan University, Stratford, New Jersey, USA; 3 Biological Mass Spectrometry Resources, Robert Wood Johnson Medical School, Rutgers, State University of New Jersey, Piscataway, New Jersey, USA

**Keywords:** cryo-EM, lectin, *Maackia amurensis*, *Maackia amurensis* seed lectin, MASL

## Abstract

*Maackia amurensis* lectins, including MASL, MAA, and MAL2, are widely utilized in biochemical and medicinal research. However, the structural and functional differences between these lectins have not been defined. Here, we present a high-resolution cryo-EM structure of MASL revealing that its tetrameric assembly is directed by two intersubunit disulfide bridges. These bridges, formed by C272 residues, are central to the dimer-of-dimers assembly of a MASL tetramer. This cryo-EM structure also identifies residues involved in stabilizing the dimer interface, multiple glycosylation sites, and calcium and manganese atoms in the sugar-binding pockets of MASL. Notably, our analysis reveals that Y250 in the carbohydrate-binding site of MASL adopts a flipped conformation, likely acting as a gatekeeper that obstructs access to noncognate substrates, a feature that may contribute to MASL’s substrate specificity. Sequence analysis suggests that MAA is a truncated version of MASL, while MAL2 represents a homologous isoform. Unlike MASL, neither MAL2 nor MAA contains a cysteine residue required for disulfide bridge formation. Accordingly, analysis of these proteins using reducing and nonreducing SDS-PAGE confirms that the C272 residue in MASL drives intermolecular disulfide bridge formation. These findings provide critical insights into the unique structural features of MASL that distinguish it from other *M. amurensis* lectins, offering a foundation for further exploration of its biological and therapeutic potential.

*Maackia amurensis* seed lectin (MASL) is a plant lectin isolated from seeds of the *M. amurensis* tree. MASL has a strong affinity for sulfated carbohydrates and α2-3–linked sialic acids (1, 2, 3). These sialic acid moieties are associated with many aspects of cell biology, including tumor progression, viral infection, immune response, and inflammation. Consequentially MASL is utilized as an agent to identify these residues on various proteins (1, 4, 5). For example, MASL can be used to target sialic acid residues on the ACE2 receptor on epithelial cells (6, 7) and the podoplanin receptor on chondrocytes (8, 9) to inhibit viral infection and arthritic inflammation (4). In addition, MASL can target sialic acid residues on receptors to inhibit mammary carcinoma (10), non–small cell lung cancer (11, 12), and acute lymphoblastic leukemia cell growth (13). In particular, MASL can target sialic acids on the podoplanin receptor to inhibit melanoma (14) and oral squamous cell carcinoma (15, 16) cell growth and motility. Indeed, MASL is being evaluated as a potential agent to inhibit oral cancer progression in an ongoing phase 1 clinical trial (NCT04188665).

In spite of their significant use, the nomenclature and protein sequences of *M. amurensis* lectins have not been clearly defined. These lectins are referred to by different monikers including MAA, MAH, MAM, and MAL2 in addition to MASL. However, distinctions between these lectins and their fundamental properties have not been elucidated (1, 15). MASL consists of subunits between 27 kDa and 36 kDa that form 72 kDa dimers revealed by reducing and nonreducing SDS-PAGE (15). A previously reported X-ray structure of MASL revealed the presence of dimers in an asymmetric unit of a crystal. While symmetry-related molecules in the crystal lattice of MASL indicated another potential dimeric interface that might result in a tetrameric structure, the biological relevance of tetramerization has not been established. Additionally, this X-ray structure of MASL lacked a portion of its C terminus, including the critical cysteine residue at position 272, preventing further analysis of its oligomerization (17).

In contrast to MASL, other *M. amurensis* lectins—including MAA and MAL2—consist of smaller subunits between 27 kDa and 31 kDa which do not dimerize in nonreducing conditions (15). Here, we utilized cryo-EM to obtain a structural model of MASL which reveals specific glycosylation sites, ion-binding sites, and an intersubunit disulfide bridge in the tetrameric protein. In addition, we directly sequenced these proteins to find that while these lectins share similar protein sequences, the cysteine that directs tetramerization of MASL subunits is absent from nondimerizing *M. amurensis* MAA and MAL2 lectins.

## Results

**Figure 1:**
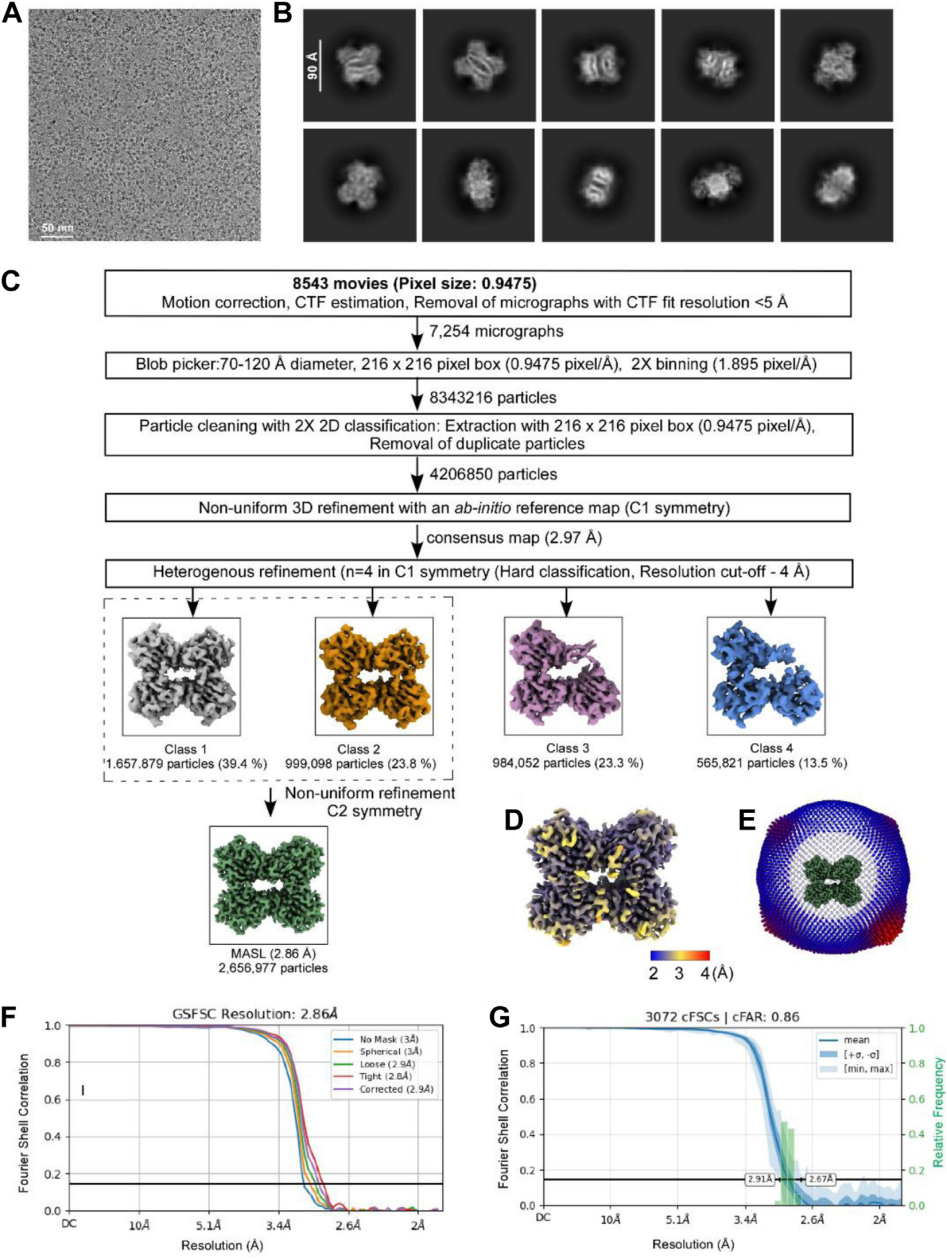
**Cryo-EM image processing workflow.***A*, micrograph of MASL particles captured at 150000x magnification. *B*, representative 2D class projections obtained in reference-free 2D classification. *C*, image processing workflow for MASL structure determination. *D*, local resolution estimates on the unsharpened map. *E*, angular distribution of MASL particles contributing to the final map. *F*, gold-standard Fourier shell correlation (FSC) plot between half maps, using a corrected tight mask, showing map resolution at 0.143 FSC. *G*, directional dependency of the map resolution, shown from a conical FSC plot representing particle orientation in the Fourier domain. MASL, *Maackia amurensis* seed lectin.

**Figure 2:**
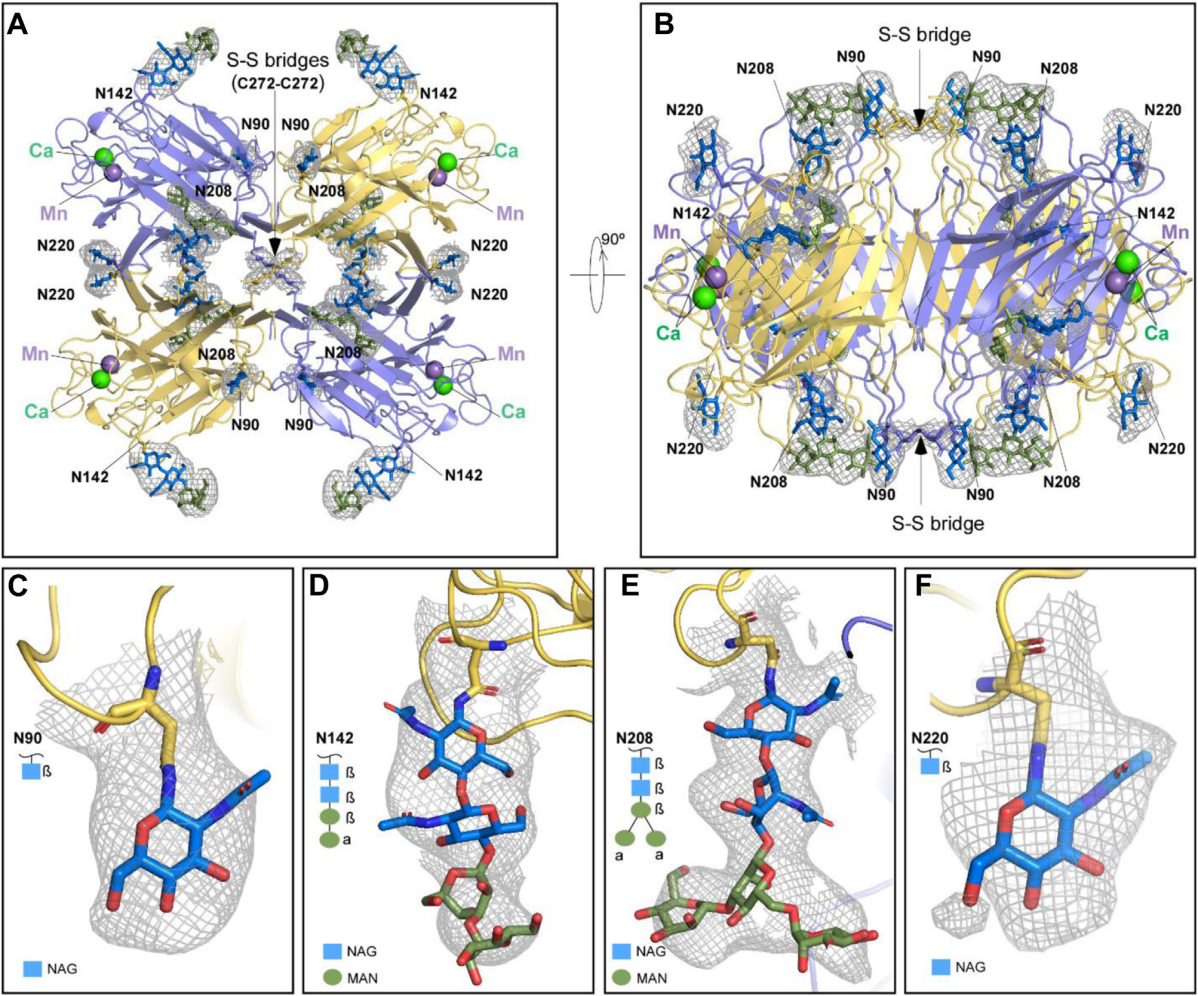
**Structure of the MASL tetramer.***A* and *B*, orthogonal views of the MASL tetramer are shown, with a *gray mesh* highlighting glycosylation sites and intersubunit disulfide linkages based on the Coulombic density map. The tetramer consists of two pairs of subunits located opposite to each other (*yellow and slate blue*), linked by disulfide bonds at C272 residues (indicated by *arrows*). Four N-linked glycosylation sites at N90, N142, N208, and N220 are depicted. Calcium (Ca^2+^) and manganese (Mn^2+^) ions are bound adjacent to the trisaccharide-binding pockets, shown in *green* and *purple*, respectively. *C*–*F*, close-up views of the Coulombic density maps for the four N-linked glycosylation sites (N90, Asn142, N208, and N220, in *yellow*). The GlcNAc and mannose sugar moieties are shown in *blue* and *green*, respectively. The mannose residues are connected through α-1,3 and α-1,6 glycosidic bonds, while GlcNAc is linked to mannose *via* a β-1,4 glycosidic bond. MASL, *Maackia amurensis* seed lectin.

**Figure 3:**
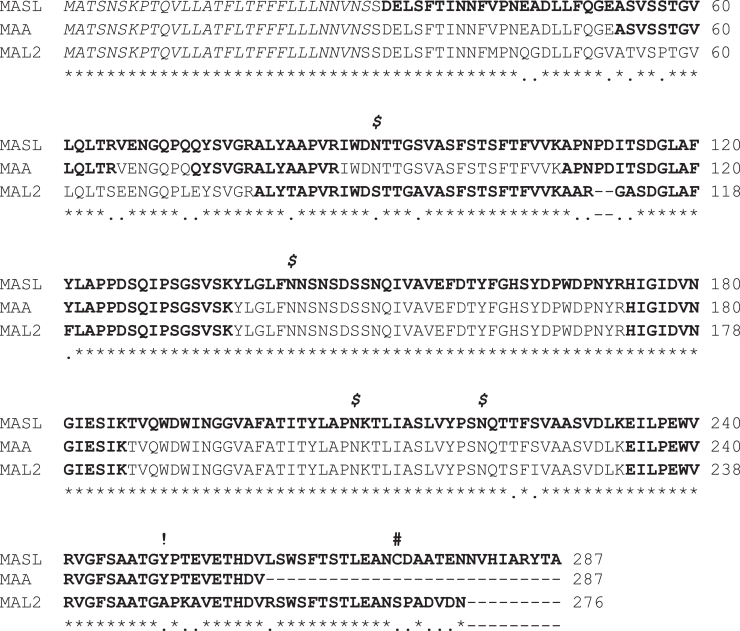
**Amino acid sequence of dimerizing and nondimerizing *Maackia amurensis* lectins.** Alignment of MASL (Sentrimed *Maackia amurensis* seed lectin) and MAL2 (Vector Labs L-1260-2 based on Yin *et al.* (15)) with MAA (EY Laboratories L-7801-1 based on Yamamoto *et al.* (33)) with sequences identified by LC-MS/MS in *bold font*. Signal peptides are *italicized*, and a cysteine or serine found near the carboxy tail of dimerizing and nondimerizing lectins, respectively, is indicated by a *hashtag*. A tyrosine at amino acid 250 in MASL and MAA, substituted with alanine in MAL2 is indicated by an *exclamation mark*. Glycosylation sites identified in MASL are indicated by an *italicized dollar sign*. Conserved residues, substitutions, and deletions are indicated by *asterisks*, *periods*, and *dashes*, respectively, with amino acids numbered as indicated.

We used cryo-EM and single-particle analysis to determine a high-resolution structure of MASL with image processing workflows outlined in Figure 1. The structure reveals a dimer-of-dimers arrangement forming the MASL tetramer, as shown in Figure 2, *A* and *B*. Dimerization of the dimers is mediated by cysteine residues at position 272, which form disulfide bridges between diagonally opposite subunits. This cysteine residue has been previously reported in the MASL sequence determined by LC-MS/MS sequencing (15) as shown in Figure 3. However, the cryo-EM structure reveals additional stabilizing interactions within the MASL tetramer including hydrogen bonds between residues T210 and Q221, as well as between the carbonyl group of Q221 and K186 residues, which further stabilize the canonical dimer interface and prevent its dissociation. Each monomer harbors a calcium and manganese ion located within the external loops, resulting in the tetrameric protein structure harboring a total of four calcium and four manganese atoms, as shown in Figure 2, *A* and *B*.

In addition to disulfide and hydrogen bond interactions, the cryo-EM structure of MASL also reveals four glycosylation sites shown in Figure 2. Three of these sites, located at the N90, N142, and N208 residues, were previously observed in an X-ray structure of MASL (17) and confirmed with additional GlcNAc and α- and β-mannose moieties as shown in Figure 2, *C*–*E*. These glycosylation sites were also identified by LC-MS/MS sequencing (15) as shown in Figure 3. However, an additional glycosylation site at the N220 residue identified by LC-MS/MS sequencing (15) shown in Figure 3 was not seen in the previous X-ray structure but was identified in the cryo-EM maps, with clear density assigned to an N-linked GlcNAc molecule shown in Figure 2*F*.

**Figure 4:**
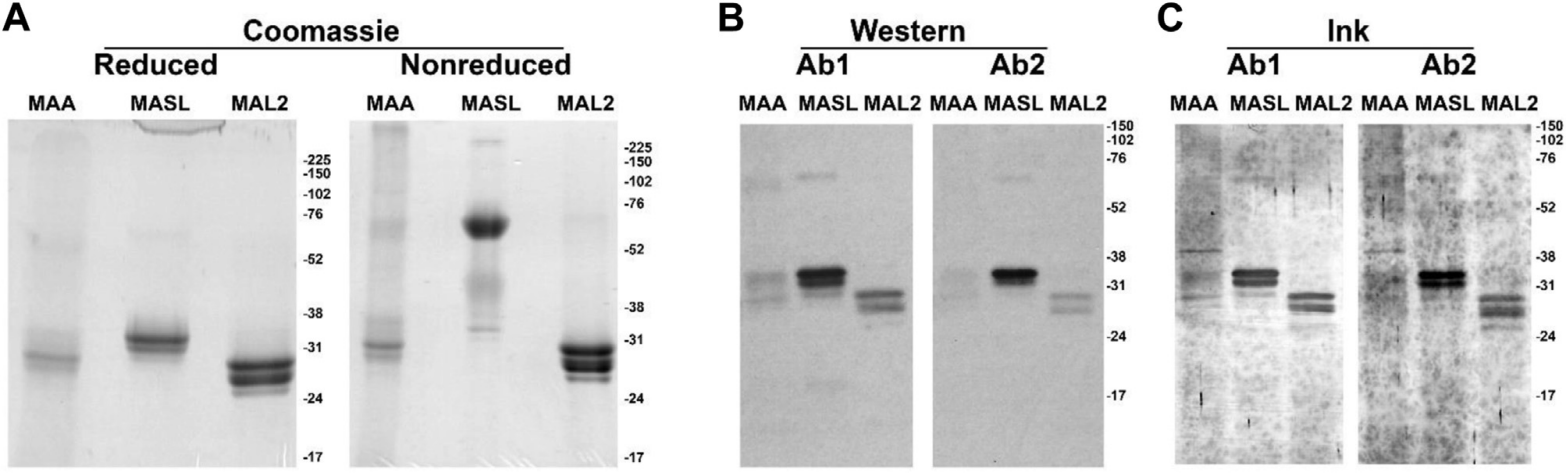
***Maackia amurensis* lectins resolved by SDS-PAGE.***A*, MAA, MASL, and MAL2 (15 ug per lane) were resolved by nonreducing and reducing 12% SDS-PAGE and visualized by Coomassie staining along with molecular weight markers as indicated. *B*, MAA, MASL, and MAL2 (1 ug per lane) were resolved by 12% SDS-PAGE with migration of molecular weight markers as indicated. Lectins were detected by Western blotting with two independent rabbit antibodies as indicated. *C*, membranes were stained with India ink after Western blotting. MASL, *Maackia amurensis* seed lectin.

Analysis of proteins using reducing and nonreducing SDS-PAGE confirms previous reports (14, 15) that MASL consists of monomeric isoforms between 36 kDa and 27 kDa on reducing gels that form a dimer of 72 kDa on nonreducing gels, as shown in Figure 4*A*. These data also confirm reports (15) that the other *M. amurensis* lectins, MAL2 and MAA, consist of monomers of 28 kDa and 27 kDa on reducing gels that do not dimerize on nonreducing gels as shown in Figure 4*A*. We developed anti-MASL antibodies in two different rabbits to study these *M. amurensis* lectins. Both antibodies recognize all three of these lectins as demonstrated by Western blot analysis shown in Figure 4, *B* and *C*. This result is consistent with the sequence homology shared among these lectins.

We used LC-MS/MS to determine how MASL, MAL2, and MAA sequences might account for their ability or failure to form dimers. These sequences indicate that a single cysteine is found at residue 272 in MASL, but no cysteine residues are found in either MAA or MAL2 as shown in Figure 3. MAA shares an identical amino acid sequence with MASL up to valine 259, where it appears to be truncated resulting in the absence of cysteine at residue 272. Interestingly, MAL2 is over 90% identical to MASL from residue 1 to valine 259 from which it diverges to about 65% identity with a serine at residue 272 instead of a cysteine as shown in Figure 3.

**Figure 5:**
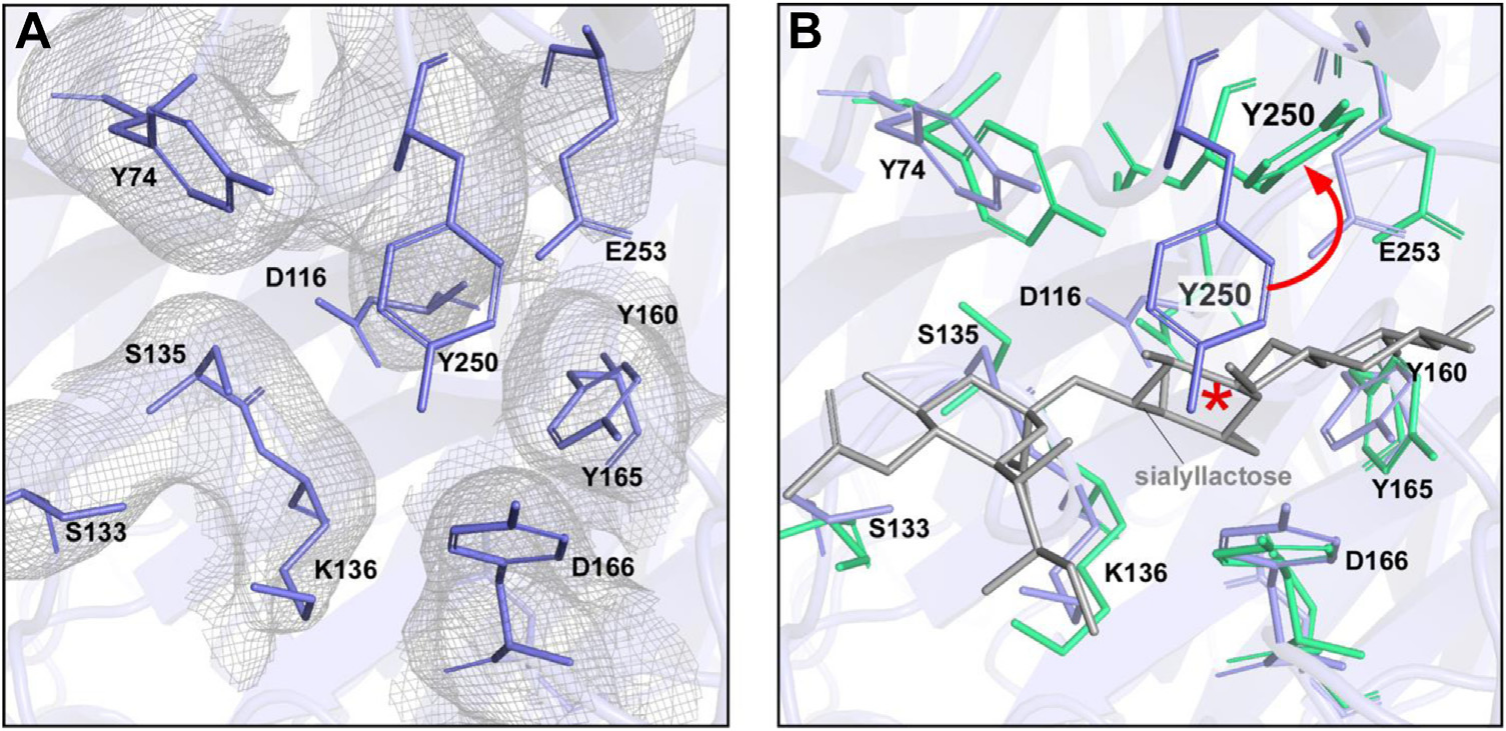
**The carbohydrate-binding site of MASL is partially obstructed by the Y250 residue.***A*, close-up view of the carbohydrate-binding site of the apo form of MASL. Key residues (shown as *slate-colored sticks*) are displayed along with the Coulomb potential density (*mesh*). *B*, superimposition of the apo MASL structure (this study) with the *glycan*-bound structure (PDB ID: 1DBN). Key residues are shown as *blue sticks* for the apo form and *green sticks* for the complex. Sialyllactose is depicted in *gray*. The flipping of the Y250 residue is indicated by a *red arrow*, and its steric clash with the *glycan* is marked by an *asterisk*. The D116 residue is omitted for clarity. MASL, *Maackia amurensis* seed lectin.

A structural comparison between previously reported substrate-bound MASL (17) and the apo form of the lectin obtained in this study shows that the overall rotameric positions of the carbohydrate-binding site residues remain largely unchanged (RMSD = 0.56 Å). However, in the apo form, the Y250 residue is rotated approximately 90 degrees, closing the carbohydrate-binding cavity and creating a steric clash with the position of sialyllactose observed in a substrate-bound MASL (17) as shown in Figure 5, *A* and *B*.

**Figure 6:**
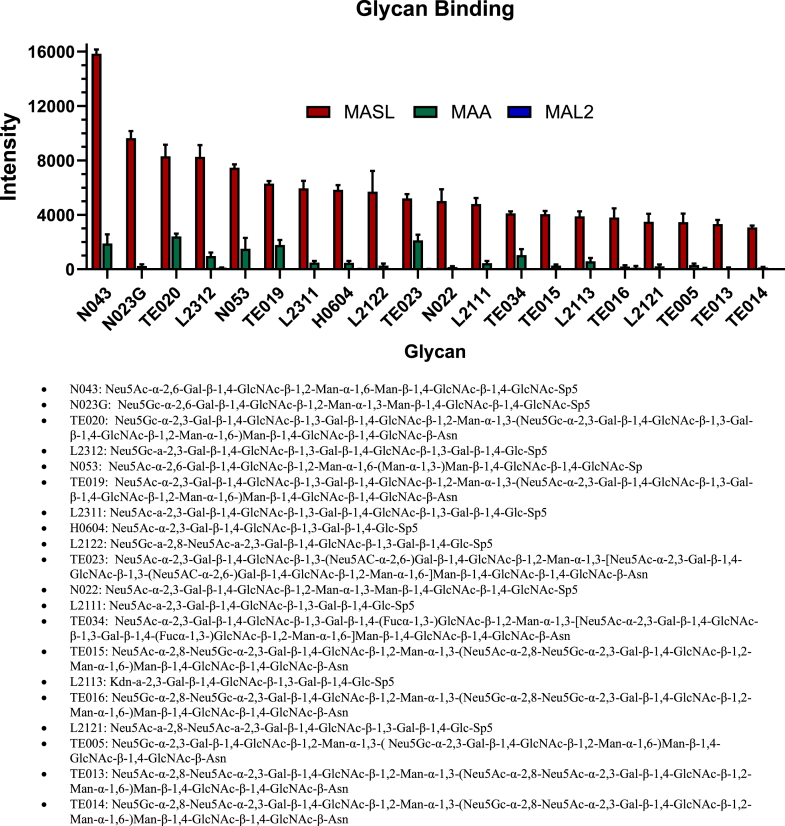
**Comparison of MASL, MAA, and MAL2 binding to glycans.** Lectins were hybridized to *glycan* arrays to compare target binding affinities. Signal intensities were normalized to positive controls with background subtracted and are shown as mean + SEM (n = 3). Twenty *glycans* with the highest affinity for MASL are shown. MASL, *Maackia amurensis* seed lectin.

We evaluated binding of MASL, MAA, and MAL2 to 300 different glycans on arrays presented in Table S3. All three lectins bound to glycans containing sialic acid moieties as expected from previous reports (1, 2, 3). However, MASL bound to its most prominent targets with a 2- to 10 fold higher affinity than MAA and a 20- to 100-fold higher affinity than MAL2. These targets include N-glycans (spots N043, N023G, N053, and N022), tandem epitopes (spots TE020, TE019, TE023, TE034, TE015, TE016, TE005, TE013, and TE014), glycolipid glycans (spots L2312, L2311, L2122, L2111, L2113, and L2121), and human milk oligosaccharides (spot H0604) included on the arrays as shown in Figure 6.

## Discussion

*M. amurensis* lectins are broadly utilized as biochemical and medicinal research agents. In particular, MASL is being evaluated as a potential agent to treat cancer and inflammatory diseases (4). However, the differences between MASL and other *M. amurensis* lectins have not been clearly described (1, 4, 15).

We confirm here that MASL consists of monomeric subunits that form dimers on nonreducing gels, while MAA and MAL2 lectin subunits do not dimerize. We sequenced each of these lectins to find a single cysteine at residue 272 in MASL, but not MAA or MAL2. These data indicate that this cysteine drives dimerization and, ultimately, tetramerization of MASL subunits.

Our sequencing revealed that MAA is a truncated version of MASL at Val 259, upstream of Cys272, the site of intersubunit disulfide bridge formation. Sequence analysis also showed that MAL2 shares over 90% identity with MASL up to Val259, after which it diverges to about 65% identity, with a serine at residue 272 instead of cysteine. These similarities are consistent with the ability of specific antibodies raised against MASL to recognize all the 3 *M. amurensis* lectins in Western blot analysis. To our knowledge, the *M. amurensis* genome has not been sequenced yet, which would help determine if MAL2 is derived from a homologous MASL gene, and if MAA is encoded by a different gene, alternate splicing, or produced by proteolytic cleavage of MASL.

Previous structural studies of MASL provided valuable insights into its architecture but lacked details about its disulfide bridges and did not elucidate the basis of its quaternary structure (17). The high-resolution structure of MASL, obtained through cryogenic microscopy and single-particle analysis, reveals the quaternary organization of the lectin in its native form. MASL homodimers (AB), known as "canonical legume dimers," are arranged into a tetrameric [AB]_2_ structure, with opposing subunits (A-A and B-B) connected by disulfide bridges formed by intramolecular disulfide bonds enabled by C272 residues. These disulfide bridges, positioned on diagonally opposite sides of the tetramer, effectively close the narrow central channel formed by the MASL subunits.

Aromatic amino acids play a crucial role in lectin–carbohydrate interactions, contributing to the adaptability and specificity of carbohydrate-binding sites. These residues interact with polysaccharide substrates through a combination of previously described (17) hydrophobic forces, van der Waals interactions, and CH-π stacking interactions seen in Figure 2. Interestingly, four out of the nine amino acids in the MASL carbohydrate-binding site are tyrosines, one of which, Y250, adopts a flipped conformation in the apo structure shown in Figure 5*B*. The position of this Y250 residue likely obstructs access to noncognate substrates in the MASL carbohydrate-binding site. Interestingly, this tyrosine residue in MASL and MAA is substituted with alanine in MAL2 as shown in Figure 3.

This type of aromatic side chain flipping has been previously observed in structural studies of *Aleuria aurantia* lectin (AAL) (18) and *Pseudomonas taiwanensis* lectin (19), where the tryptophan side chain interferes with glycan binding. Similarly, the position of the Y250 residue in MASL likely acts as a gatekeeper, preventing noncognate substrates from accessing the carbohydrate-binding site. The flipping of Y250 is presumed to occur as part of an induced fit mechanism, in which the binding pocket reshapes itself to enhance molecular recognition. Interestingly, the Y250 residue of MASL is substituted with an alanine in MAL2 shown in Figure 3, which may influence the substrate specificity of MAL2 compared to MASL shown in Figure 6.

While the overall tetrameric arrangement of MASL resembles other tetrameric lectins, such as soybean agglutinin (20) and *Phaseolus vulgaris* phytohemagglutinin-L (21), the presence of disulfide bridges adds a unique stabilizing feature to its structure. To the best of our knowledge, this characteristic has not been described for any other tetrameric lectin. Taken together, data from this study reveals unique properties of MASL that might be relevant to its utilization as a research, diagnostic, and medicinal compound.

## Experimental procedures

### Cryo-EM grid preparation

Natural MASL purified from *M. amurensis* seeds (Sentrimed) was used for this study. To prepare samples for cryo-EM, MASL was dissolved in a buffer containing 20 mM Tris (pH = 7.9), 100 mM NaCl, 0.1 mM MnCl_2_, and 0.1 mM CaCl_2_ at a final concentration of 3 μM as a tetramer. MASL solution (2.5–3 μl) was applied to negatively glow-discharged 300 mesh Ultrafoil 1.2/1.3 grids (Quantifoil). Grids were blotted with Whatman Grade 595 filter paper in a Vitrobot Mark IV (Thermo Fisher Scientific) for 5 s at 4 °C and 95% humidity, vitrified in liquid ethane, and the clipped and mounted on the autoloader cassette.

### Data acquisition and image processing

Single-particle cryo-EM data for MASL was collected at the Integrated Structural Biology Shared Resources, Thomas Jefferson University, using a Glacios cryo-transmission electron microscope (Thermo Fisher Scientific) operating at 200 kV. Movies were acquired at a nominal magnification of 1,500,00× in fast acquisition aberration-free image shift mode, resulting in a pixel size of 0.9475 Å with a Falcon 4 direct electron detector and a C2 aperture of 50 μm. The setup included stigmation and coma correction on a crossline grating grid. An exposure time of approximately 8.5 s yielded a total electron dose of 60 e^-^/Å^2^, distributed across 40 frames. Single acquisitions at the center of the foil hole were enforced. Data collection was performed using EPU 3.6 software (https://www.thermofisher.com/us/en/home/electron-microscopy/products/software-em-3d-vis/epu-software.html) with defocus values ranging from −0.3 to −1.2 μm. Focusing and drift measurements were performed on a foil region 0.65 μm adjacent to the foil hole, repeated after centering and once per grid square, respectively, with a drift threshold of 0.4 nm/sec. Cryo-EM image acquisition parameters are summarized in Tables S1 and S2.

The movie stacks were processed using CryoSPARC 4.4 (https://cryosparc.com) (22). Frame alignment, motion correction, gain normalization, and dose weighting were performed with the patch motion correction module, and contrast transfer function (CTF) values were estimated with CTFFIND4 (23). Micrographs with ice contamination, ethane artifacts, or a CTF fit resolution worse than 5 Å were excluded. MASL particles were picked using a circular blob picker with diameters ranging from 70 to 120 Å and were downsampled 2 × during 2D classification. Particles smaller than 40 Å, identified as duplicates, were discarded during 2D classification. Particles lacking tetrameric organization or missing subunits were separated *via* heterogeneous refinement.

A 2.9 Å resolution map of the MASL tetramer was obtained from 2,656,977 particles and used for *ab initio* model building following pixel size calibration with a low-pass filtered X-ray map of the crystal structure (PDB: 1DBN) (17). The pixel size of the MASL cryo-EM map was refined to 0.93 Å which yielded the maximum cross-correlation coefficient with the X-ray map in UCSD Chimera (24). The reported resolutions of the cryo-EM maps are based on the Fourier shell correlation 0.143 criterion. Directional isotropy of the 3D reconstruction along three orthogonal axes was assessed in CryoSPARC, while angular distribution and local resolution plots were generated in ChimeraX (25).

### Model building and structure refinement

Four molecules of the MASL from a previously reported crystal structure (PDB ID: 1DBN) were docked into the tetrameric Coulombic map resolved up to 2.9 Å, and the polypeptide chain was morph-fitted in Coot 0.9.8.5 (26). The local density fit of MASL polypeptide (residues 30–273) were improved over an iterative process of model fitting in Coot (https://www2.mrc-lmb.cam.ac.uk/personal/pemsley/coot), followed by further fine-tuning using real-space refinement in PHENIX (https://www.phenix-online.org) (27). Real-space refinement was carried out with secondary structure and Ramachandran restraints. The Coulombic density for the N-terminal 29 residues of MASL was absent, similar to the crystal structure. The cryo-EM map resolved five additional residues at the MASL’s C terminus (residues 269–273), including Cys272, which is involved in an intersubunit disulfide bond. The cryo-EM map also lacks density for the C-terminal 14 residues (residues 274–287), which form an alpha helix according to an AlphaFold-2 predicted model. Comprehensive model validation of the MASL refined model was carried out with PHENIX and the PDB validation server (https://validate-rcsb-2.wwpdb.org/). Map-to-model correlation coefficient and Fourier shell correlation plots were obtained in PHENIX. Figures were generated with PyMOL (https://www.pymol.org) and ChimeraX (28).

### SDS-PAGE and LC-MS/MS

MASL (Sentrimed), MAA (EY Laboratories #L-7801–1), and MAL2 (Vector Labs #L-1260–2) were resolved on a 12% SDS-PAGE gel (15 ug/lane) in loading buffer (2% SDS, 10% glycerol, and 0.05% bromophenol blue in 62.5 mM Tris–HCl pH6.8) with (reducing) or without (nonreducing) 10% β-mercaptoethanol, and stained with Coomassie Brilliant blue R 250 (Sigma-Aldrich #CI42660). Coomassie stained bands were excised from SDS-PAGE reducing gels and sequenced by LC-MS/MS as described (15).

### Western blotting

Rabbits were immunized with full-length native MASL (Sentrimed) by Proteintech (Proteintech #90001). Serum from these rabbits was affinity-purified with MASL (Pierce MicroLink Peptide Coupling Kit, Cat No: 20485) as previously described (29, 30). MASL, MAA, and MAL2 were resolved by 12% SDS-PAGE (1 ug/lane) in loading buffer (2% SDS, 10% glycerol, and 0.05% bromophenol blue in 62.5 mM Tris–HCl pH6.8, 10% β-mercaptoethanol), and transferred to immobilon-P membranes (EMD Millipore #IPVH00010). Membranes were then incubated with affinity purified anti-MASL primary antiserum at a 1:500 dilution. Primary antiserum was recognized by secondary antiserum specific for rabbit (Cell Signaling #7074) at a 1:5000 dilution and detected using enhanced chemiluminescence (Thermo Fisher Scientific 32209) as described (15). Gels were stained with Coomassie, and membranes were stained with India ink to verify efficient loading and transfer after blotting.

### Glycan microarray

Microarray screening studies were performed by RayBiotech as previously described (31, 32) with some modifications. MASL, MAL2, and MAA lectins were biotinylated and hybridized (30 ug/ml) to Glycan Array 300 arrays (RayBiotech) which contain 300 triplicated synthetic glycans along with biotinylated protein positive controls and empty negative controls. Lectin binding was detected by incubation with Cy5 equivalent dye-conjugated streptavidin and visualized with a GenePix 4000B microarray laser scanner equipped with GenePix Pro version 7.2 software (https://genepix-pro.software.informer.com/7.2) (Molecular Devices). Mean signal intensities were normalized to positive controls and analyzed using RayBio GA-Glycan-300-SW Analysis software (https://www.raybiotech.com/glycan-array-300-ga-glycan-300) (RayBiotech).

## Data availability

Cryo-EM maps and atomic coordinates of MASL were deposited in the Electron Microscopy Data Bank under accession code EMD-47565, and in the Protein Data Bank under accession code 9E6H.

## Supporting information

This article contains supporting information.

## Conflict of interest

The authors declare the following financial interests/personal relationships which may be considered as potential competing interests: G. S. G. has intellectual property and ownership in Sentrimed, Inc; D. T. acts as consultant and has ownership in Sentrimed, Inc; and C. J. H., R. E. N., and A. C. Y. have ownership and received financial support from Sentrimed, Inc which is developing agents including MASL that target podoplanin to treat diseases including cancer and arthritis. The other authors declare that they have no conflicts of interest with the contents of this article.
