# Supplementary material for: Maackia amurensis seed lectin structure and sequence comparison with other M. amurensis lectins
Source: J Biol Chem. 2025 Mar 28;301(5):108466. doi: 10.1016/j.jbc.2025.108466 (PMC12059335; doi:10.1016/j.jbc.2025.108466)
Supplement: Table S1 and S2 [file mmc1.docx]

| **Data Acquisition** | |
| --- | --- |
| Electron Microscope | Thermofisher Glacios |
| Data collection Software | EPU 3.6 |
| Acquisition type | Fast acquisition (AFIS) |
| Voltage (kV) | 200 |
| Detector | Falcon 4 |
| Magnification | 150000 |
| Condenser (C2) Aperture Size (μm) | 50 |
| Objective Aperture | None |
| Align | Yes |
| Delay After stage Move (sec) | 7 |
| Autofocus recurrence | After centering |
| Drift Measurement (Threshold in nm/s) | Once per grid square (0.4 ) |
| Number of images per hole | 1 |
| Grid type | 300 mesh UltraFoil 1.2/1.3 μm |
| Pixel Size (Å) – Nominal (Calibrated) | 0.9475 (0.93) |
| Focus range (μm) | -0.3 to -1.2 |
| Total electron dose (e/Å^2^) | 60 |
| Number of frames,  Dose per frame (e/Å^2^) | 40, 1 |
| Number of movies collected | 8543 |
| Number of micrographs used | 7254 |
| **Image Processing** | |
| Program | CryoSPARC 4.4 |
| Number of particles picked | 8343216 |
| Number of particles  (After 2D classification) | 4206850 |
| Number of particles  (After 3D classification) | 2656977 |
| Symmetry imposed | C2 |
| Map Resolution FSC 0.143 (Å) | 2.84 |
| EMDB ID | 47565 |

**Table S1: Summary of Cryo-EM data collection and Single Particle Analysis.**

| **Refinement** | |
| --- | --- |
| Initial Model used (Residue range) | 1DBN (30-269) |
| Nonhydrogen atoms | 8106 |
| Protein residues | 978 |
| Ligands (NAG, Mannose, Ca, Mn) | 20, 24, 4, 4 |
| RMS Deviation (Bonds), Å | 0.002 |
| RMS Deviation (Angle), ° | 0.635 |
| Ramachandran Plot |  |
| Preferred (%) | 98.35 |
| Allowed (%) | 1.65 |
| Outliers (%) | 0.00 |
| Model to map Correlation Coefficient (­CC_mask_) | 0.75 |
| **Validation** | |
| Clash-score | 3.06 |
| MolProbity score | 1.1 |
| Rotamer outliers (%) | 0.00 |
| PDB ID | 9E6H |

**Table S2: Model refinement and validation statistics.**
